# Conceptual and practical challenges for implementing the communities of practice model on a national scale - a Canadian cancer control initiative

**DOI:** 10.1186/1472-6963-10-3

**Published:** 2010-01-05

**Authors:** Colene Bentley, George P Browman, Barbara Poole

**Affiliations:** 1 Cancer Control Research, B.C. Cancer Agency, 675 W. 10th Avenue, Vancouver, B.C., Canada; 2 Cancer Control Research, B.C. Cancer Agency, Vancouver Island Centre, Victoria, B.C., Canada; 3 School of Population and Public Health, University of British Columbia, Vancouver, B.C., Canada

## Abstract

**Background:**

Cancer program delivery, like the rest of health care in Canada, faces two ongoing challenges: to coordinate a pan-Canadian approach across complex provincial jurisdictions, and to facilitate the rapid translation of knowledge into clinical practice. Communities of practice, or CoPs, which have been described by Etienne Wenger as a collaborative learning platform, represent a promising solution to these challenges because they rely on bottom-up rather than top-down social structures for integrating knowledge and practice across regions and agencies. The communities of practice model has been realized in the corporate (e.g., Royal Dutch Shell, Xerox, IBM, etc) and development (e.g., World Bank) sectors, but its application to health care is relatively new. The Canadian Partnership Against Cancer (CPAC) is exploring the potential of Wenger's concept in the Canadian health care context. This paper provides an in-depth analysis of Wenger's concept with a focus on its applicability to the health care sector.

**Discussion:**

Empirical studies and social science theory are used to examine the utility of Wenger's concept. Its value lies in emphasizing learning from peers and through practice in settings where innovation is valued. Yet the communities of practice concept lacks conceptual clarity because Wenger defines it so broadly and sidelines issues of decision making within CoPs. We consider the implications of his broad definition to establishing an informed nomenclature around this specific type of collaborative group. The CoP Project under CPAC and communities of practice in Canadian health care are discussed.

**Summary:**

The use of communities of practice in Canadian health care has been shown in some instances to facilitate quality improvements, encourage buy in among participants, and generate high levels of satisfaction with clinical leadership and knowledge translation among participating physicians. Despite these individual success stories, more information is required on how group decisions are made and applied to the practice world in order to leverage the potential of Wenger's concept more fully, and advance the science of knowledge translation within an accountability framework.

## Background

### The need for collaboration

Western health care systems are challenged to provide accessible, relevant, and integrated services. To confront these challenges, publicly-funded health systems must find effective ways to increase inter-professional and inter-regional collaboration so that expertise can be enhanced, disseminated, and more effectively translated into practice.

Recently funded by the federal government, the Canadian Partnership Against Cancer (CPAC) is an innovative response to the need for forward nation-wide strategic planning to help the Canadian population meet the challenges that will accompany the growing burden of cancer. Because cancer program delivery, like the rest of health care in Canada, is the responsibility of different jurisdictions, such as provincial governments, national solutions rely on fostering inter-provincial collaborations. The Partnership has thus embarked on an ambitious agenda that recognizes the need to develop both technological and social platforms to facilitate and nurture sustainable pan-Canadian collaborative efforts. In this context, the Partnership has been exploring the utility of Etienne Wenger's "communities of practice" (CoP) concept. The Partnership's CoP Project was conceived to identify and evaluate the effectiveness of communities of practice to enhance collaboration and knowledge translation in cancer control across Canada. Here we describe what we have learned from preliminary stages of the CoP Project, and offer further insights into this promising model of collaboration.

Since the early 1990s, CoP-type structures have been implemented primarily in the business and development sectors; however, their application to health care is a more recent consideration. In this paper, we identify the potential application of Wenger's concept to health care settings, based on a literature review and our experience with the CoP Project. Our aim is to gain a more precise understanding of communities of practice, including their capacity as a form of social infrastructure to further knowledge translation and sustain collaborative efforts across traditional health care jurisdictions and disciplines. The terms *collaboratives *and *groups *are used generically throughout the paper to denote interactive work groups.

### The communities of practice concept

"Communities of practice are groups of people who share a concern, a set of problems, or a passion about a topic, and who deepen their knowledge and expertise in this area by interacting on an ongoing basis"[1].

This definition of communities of practice captures the three structural elements of a community of practice, namely, its *domain*, *practice*, and *community*. It is worth elaborating on these three elements in the context of Wenger's other writings to understand this concept more fully.

The *domain *of a community of practice refers to the common "concern, set of problems, or passion about a topic" that all members share and around which they organize. It is the area of members' overlapping expertise, and it provides the initial centripetal pull uniting individuals who might otherwise work in separate organizational units, disciplines, or locations. Members have an abiding concern about their common topic, and collaborate to solve problems relating to it. They are also "passionate" about it. Wenger observed that, "If members don't feel personally connected to the group's area of expertise and interest...they won't fully commit themselves to the work of the community" [2].

According to Wenger, communities of practice form serendipitously when members recognize their common domain. Communities of practice are thus "fundamentally informal and self-organizing" entities, and can "benefit from cultivation" [2], which is the agricultural metaphor employed extensively by Wenger. The largely organic and non-prescriptive origins of these groups provide the conditions for members to participate creatively in practice [1].

*Practice *is the second structural element of the communities of practice concept, and represents the basic body of knowledge the group shares and builds. Members of a community of practice aim to "deepen their knowledge and expertise" on a topic by learning from each other. More specifically, members learn from peers and through practice, which is why members of a community of practice are *practitioners*. Here Wenger draws from earlier work with Jean Lave on guild systems and apprenticeship models, where learning and its application take place in the same setting [3]. "Through its practice--its concepts, symbols, and analytic methods--the community operates as a living curriculum," according to Wenger *et al *[1].

Communities of practice value both tacit and explicit forms of knowledge. They recognize the need to complement individuals' intuitions, perceptions, and vernacular knowledge with the more explicit concepts of an evidence-based paradigm. "The knowledge of experts is an accumulation of experience--a kind of 'residue' of their actions, thinking, and conversations that remains a dynamic part of their ongoing experience" [1]. In this respect, communities of practice are social learning structures: they are open venues of exploration, "where it is safe to ask hard questions and speak the truth" and where members "develop the habit of consulting each other for help" [1].

The third structural element of a community of practice is the idea of *community*, although scholars have also identified it as the most troublesome to reconcile. Community denotes a set of interpersonal relationships arising out of people's mutual engagement in learning through practice. Significantly, these relationships do not indicate members' specific roles per se, but refer to their reciprocal ties of accountability, dependency, trust, and communication. These bonds of connectivity, together with the community's negotiated meanings and shared expertise, can be thought of as providing the cohesion that lends a community of practice its identity and coherence over time. Ideally, a community of practice engenders a strong sense of identity and belonging across members, even those who do not interact regularly or face-to-face. To regard collaborative groups as communities underscores their deeply normative character, which specifies the behaviours, meanings, and routines acceptable to each group.

Wenger *et al *argued that when the three structural elements function well together, they "make a community of practice an ideal *knowledge structure--*a social structure that can assume responsibility for developing and sharing knowledge" (emphasis in original) [1]. Here, knowledge and sociability are not just linked, but are mutually informing, and the dynamic between them generates the specific know-how the group builds, shares, and manages.

## Discussion

### Potential value of Wenger's concept

Wenger's concept has gained its share of champions among practitioners and scholars in the business, development, and more recently, health sectors. A recognized strength is its insistence on collaboration as a practice-based learning process. Literature on communities of practice has almost unanimously supported the view that learning by doing is more effective than didactic instruction for adults working in today's knowledge economy [4-9]. In the health sector, authors of several studies proposed that health workers and organizations interrelate learning, practice, and peer input to enhance individual and institutional performance [5,10-13]. Parboosingh, for example, argued that such interrelationships provided continuing education for physicians through ready access to a wealth of data, experiences, and mentors; he also suggested that through the dynamics of shared learning, health professionals become more reflective about their practice when they subject it to peer-based critical scrutiny [5]. Parboosingh proposed that physicians resemble Donald Schön's "reflective practitioner" when their knowledge is embedded in practice, informed by a body of evidence-based research, and shaped by the process of rethinking tacit assumptions [5]. CoP-type structures thus acknowledge the need for knowledge workers to be skilled at appraising and integrating various types of knowledge into practice and, ideally, to be forthcoming about it among peers [14,15]. Reflection on practice is also suggestive of the need for workers to contend with the medical, economic, political, and socio-cultural understandings that inform the work they do [5,10-12,16].

Because Wenger's concept does not privilege research evidence over experience-rooted knowledge, communities of practice can be venues for bridging traditional rifts in the health sector between research and practice, and among disciplines [12,17-19]. This is because a community of practice is an interpretive community. Wenger has drawn explicitly from Stanley Fish's theory of interpretive communities in the practice of literary and legal hermeneutics. Fish proposed that trained professionals determine the constraints on interpretation in their respective fields [20]. For Wenger, communities of practice are interpretive communities because they create their own understandings of the practice-world through the ongoing process of interpreting that world, thus producing the very knowledge that is held in common. The production of common knowledge through professional practice is echoed by Kerfoot: "When physicians, nurses, and clinicians are willing to come together in best practice teams, they are a learning community that has developed the relations that will make them effective" [18].

Notwithstanding the theoretical predictions, whether or not communities of practice are effective forms of collaboration remains an open question. Authors of a 2008 Canadian study found that extant literature on Wenger's concept gives little evidence of its usefulness beyond the level of description [21]; this observation is echoed throughout the communities of practice literature and lays the groundwork for future research in the field. Yet the Canadian study, like other studies, also recognized within the literature a burgeoning account of the inherent value of communities of practice for both the individual and the organization [11,12,19,21,22]. Among managers and employees alike, communities of practice are a compelling form of infrastructure because collaboration is organized around knowledge domains, not products or markets; this appeals to an increasingly educated workforce operating in settings where innovation is valued.

A further appeal of communities of practice is that they foster an egalitarian ethos by placing knowledge management in the hands of practitioners, not managers [23]. Collaborative learning has been shown to increase members' sense of ownership and autonomy when they take responsibility for setting work agendas, with ownership and autonomy identified as important factors for success in collaborative work [7,13,24-26]. Enhanced collaborative learning has also been shown to improve job satisfaction [4,5,13,25], which in turn can lead to potential increases in recruitment and retention of employees as high-value assets in learning organizations [18,27]. Conversely, Pereles *et al*. observed that when small work groups are unable to take charge of learning, membership and personal growth wane [4]. Even groups that collaborate solely through email networks value the communal nature of knowledge development and sharing [28,29]. Moreover, while evidence is only emerging on the success of heath sector communities of practice to implement the know-how they develop [30], in 2003 members of the Surgical Oncology Program (SOP) at Cancer Care Ontario endorsed Wenger's model as a means to facilitate quality improvements in cancer care [31]. Subsequent pilot tests of the model in Ontario regional cancer surgery settings revealed above-average ratings of physician satisfaction and levels of compliance with guidelines in clinical environments [13,26].

### Limitations of Wenger's concept

Wenger's model has drawn its share of detractors. Critics have focused mainly on the internal dynamics of his concept of communities of practice to argue that the complexity of social interaction within them is inadequately recognized. The application of social science theory in particular to Wenger's concept has been a fruitful avenue of inquiry, because it has helped illuminate barriers to knowledge development in CoP-type structures, as well as identify key strategies to overcoming such barriers [13,32]. Specifically, it has helped researchers to 1) gain a critical perspective on how power relations may operate in professional collaboration, and 2) better understand where Wenger's concept lacks clarity, particularly around the notions of community and the so-called natural formation of CoPs.

#### Limitation 1: The role of power

One prominent strand of critical inquiry from the social sciences has built on Michel Foucault's notions of *power*, *norms*, and *discourse *to consider the social forces at work in collaborative groups [12,14,33-36]. Foucault argued that power is not simply repressive or hierarchical, but is generated through a range of social expressions, or discourses, that circulate through our everyday social practices, investigations, talk, and writing. He proposed that determining *how *a particular topic is talked about gives a person or persons power over that topic; in fact, it brings that topic into being, thus creating the very norms by which it becomes known to others.

As social structures that leverage knowledge, communities of practice can be viewed as places where power, discourse, and norms operate. As interpretive communities, CoPs influence how knowledge is made, utilized, and valued; for this reason, they can be prone to reproducing the status quo. In their study of multi-agency communities of practice in health and social services for seniors, Gabbay *et al*. noted that the type of evidence utilized by these groups was strongly linked to the agenda and power that individuals held within the group at the time, and that many professionals' contributions to discussion could be seen as promoting parochial professional interests or disciplines [14]. Likewise, Marshall's ethnographic study of collaborative problem-solving in the telecommunications sector showed that technicians repeatedly and often subconsciously leveraged their authority over others by asking them to trust their expertise, not query it [34]. Authors of these studies demonstrated that collaborative groups are not themselves unsituated venues of professional learning and identity formation when social or professional norms are reproduced through them. For this reason, it can be difficult for alternative expressions of practice-based knowledge to be articulated and accommodated in collaborative groups [15,34,37,38]. Certainly, Wenger has acknowledged that communities of practice can become cliques, and that strong ties of membership can create barriers to outsiders. He has said surprisingly little, however, about the processes by which decisions about practice are made, and by which new medical or socio-cultural understandings make their way into the community and inform its practice.

#### Limitation 2: Lack of clarity of CoP concept

Critics have also noted that the communities of practice concept lacks clarity [36,39]. Most recently, Li *et al*. argued that the focus of Wenger's concept has diverged significantly over time, from being a theory of learning and identity development to being a tool for organizations to manage knowledge workers. They also argued that Wenger *et al*.'s comparison of CoP groups to other work structures like project teams is often vague and contradictory [39]. Our analysis revealed two additional reasons why Wenger's concept often lacks clarity: 1) communities of practice are equated with natural phenomena, like plants, when in fact they are human constructions; and 2) communities of practice are claimed to populate all domains of social experience. Together, these aspects of Wenger's concept have clouded rather than clarified understandings of what communities of practice really are

##### Doing what comes naturally

Wenger has compared communities of practice to organic phenomena, thus aligning *made *structures with *natural *ones. Nowhere is this more evident than in his investment in metaphors of nature to explain how communities of practice work. While reasoning by metaphors can stimulate new thinking on a topic, it can also spread misperceptions of the topic. According to Wenger *et al*., communities of practice are like "gardens" that "benefit from cultivation": managers can "grow communities of practice from seed" if they "till the soil, pull out weeds, add water during dry spells, and ensure that [their] plants have proper nutrients" [1,2]. They proposed that "even though communities [of practice] are voluntary and organic, good community design can invite, even evoke, aliveness."[1] The difficulty here is that figurative language--words such as "aliveness," "cultivation," "gardens," and so forth--stands in for and thus obscures the dynamics of real social processes operating within communities of practice that social science theory has sought to better understand. It suggests that complex human interaction is a simply matter of doing what comes naturally. This interpretation of social interaction as natural behavior has been discredited by social science theory of the last half of the twentieth century, because it casts ideologically vested or culturally constructed phenomena as part of the natural order of things.

Wenger also proposed that individuals coalesce into communities of practice by following their inclinations. He argued that professionals "tend to know when and if they should join [a community of practice]. They know if they have something to give and whether they are likely to take something away. And members of an existing community, when they invite someone to join, also operate on a gut sense of the prospective member's appropriateness for the group" [2]. Here, operating by gut senses and a process of member-making somewhat akin to natural selection can once again make communities of practice susceptible to reproducing the status quo. More importantly perhaps, it is difficult to square this view of the non-analyzing practitioner, that is, one who simply knows the appropriateness of certain things, with that of the reflective practitioner who is open to alternative ways of knowing and doing. It is also difficult to reconcile with Wenger's belief that individuals are motivated to join collaborative groups in order to develop a professional identity and fit in with their peers, given that instincts and motives are separate orders of mind.

Authors of empirical studies on communities of practice in health and business sectors have challenged Wenger's characterization of CoPs as self-organizing organic structures. They confirmed that work-based collaborations often require more financial support for things such as face-to-face meetings and facilitation than he acknowledged [4,14,19,24,26,40]. Studies have also shown that institutional support may also involve coordinating group activities more purposively with stakeholder objectives [24,25,30], and putting leadership in the hands of experts or champions, [24] rather than distributing it, as Wenger urged.

##### The idea of community

Wenger's concept also lacks clarity around the idea of community. Significantly, he has used the term *community *over, say, *network *or *team *precisely because he wished to endow communities of practice with ties of belonging, shared meanings and repertoires, and passion. For Wenger, these are the ties that bind heterogeneously-composed groups to a common practice and identity, which are reproduced through members' ongoing interpretation of their practice-world. According to Wenger, project teams and informal networks rely on specific goals and mutual need respectively to hold them together, whereas members of a CoP are united by an ongoing commitment to practice, a galvanizing collective consciousness, and a strong sense of belonging [2]. In *Communities of Practice*, he drew specifically on Benedict Anderson's seminal work on imagined communities and the rise of nationalism [41] to explain how CoP members envision and cultivate their commonality with others whom they may never meet face-to-face. In essence, Wenger's communities are cultural groups in ways that networks and teams are not. However, Wenger has objected to associating his notion of community with culture or prescriptive behavior [42], and has preferred instead to emphasize their creative possibilities. However, what else are rituals, special attachments, and a shared history if not the cultural markers of collective life? One can appreciate Wenger's wish to avoid the culture wars in his analysis, but he seems to want the benefits of communitarianism without its attendant baggage.

The idea of community also lacks precision in Wenger's concept because he dilutes it. Communities of practice, he claims, are everywhere, and he has included workers, disaster relief groups, garage bands, study groups, and recovering alcoholics among his many examples of them. Moreover, communities of practice can be small or large in size, and exist in the public and private domains. Given their astonishing variety and prominence, where does the category of community of practice begin and end? In what sense are paid work groups the same thing as social communities, or even families? For example, families have their own private rituals and terms of endearment, but genetics most often defines the space of membership within them, not culture. Moreover, the fact that families are chiefly genetic and legal entities means that they are not as fluidly joinable or legally breakable as are communities of practice. In a similar vein, employees may care about the work they do, but to insist that they care deeply or passionately about it is to overly sentimentalize their commitments to the practice-world. Imputing a sentimentalized solidarity to groups risks diminishing what may well be knowledge-workers' intellectual, principled, or pragmatic motives to participate in a community and identify with it [12,24,36]. Paradoxically, Wenger's many examples at once claim too much on behalf of communities of practice by saying they are strongly affective entities, and too little, because their ubiquity renders them unremarkable. Joanne Roberts, in her critique of Wenger's concept, considered the many differences between small and multinational organizations Wenger offers as examples of communities of practice, and asked: "Is it really possible to apply exactly the same principles to these two [types of] communities of practice?"[36].

### Knowledge translation and implementation

From Wenger's definition of a community of practice, it is clear that the main purpose of a CoP is to build and share knowledge related to practice. If this is the case, what, then, do we make of groups faced with the task of coordinating human activity or implementing change, in addition to sharing practice-based know-how? Strictly speaking, is it a misnomer to call them communities of practice, because they go beyond enhancing collective know-how to also include collective action? Consider two scenarios from studies involving collaborative groups in health care. The first is D'Amour *et al*.'s 2008 study on the effectiveness of collaboration among professionals in the Canadian province of Quebec. Collaboration involved linking hospital care with primary care services around the early discharge of newborns from hospital [43]. Members of these groups were committed to their practice of perinatal care, and shared information in order to improve their practice. D'Amour *et al*. developed indicators to measure the groups' intensity of collaboration and link it to clinical outcomes. They argued that it is important to understand how groups manage the responsibility of incorporating novelty into action, so as to learn how to leverage it. D'Amour *et al*.'s interest in the *processes *of decision making around new practices augments Wenger's model, and the study's groups are called collaboratives, not communities of practice.

The second scenario is CHAIN (Contact, Help, Advice and Information Network for Effective Health Care), which is a large informal online community of practice. In the UK, CHAIN's function is to share information and experience among professionals interested in evidence-based health care, and it has been highly effective in giving participants access to professionals from different organizations and disciplines, and in linking novices with experienced practitioners [28]. However, unlike members of D'Amour *et al*.s collaboratives, CHAIN's membership is not required to make group decisions, coordinate compliance with guidelines or objectives, nor to take collective action: for members of the UK CHAIN, collaboration does not entail joint deeds. Is CHAIN a better test case for Wenger's concept than D'Amour *et al*.'s groups? Does it matter if enhanced know-how--or improved practice--occurs at the individual, group, or system levels? The answers to these questions may determine the extent to which Wenger's model of collaboration is sufficient for moving thought into action that is associated with professional learning and knowledge translation. They may also determine how the effectiveness of Wenger's groups is to be identified and measured.

### Communities of practice: the Canadian health care context

Under the auspices of the Canadian Partnership Against Cancer (CPAC), the CoP Project is currently addressing questions regarding the effectiveness of Wenger's model in facilitating practice-based learning. The initiation of the CoP Project was prompted by the need for an accountability tool for a strategy aimed primarily at facilitating the development of social collaborative structures to enhance knowledge translation in cancer control. These collaborative structures span traditional jurisdictions that ordinarily do not interact at a national level in Canada. The CoP Project's main objective is to design and test a tool for evaluating the performance of communities of practice in achieving their own stated objectives and advancing the agendas for which they implicitly exist. The secondary objective is to create a workbook similar to standard operating procedures, which communities of practice can use to guide data collection in order to assess their own performance over time. The products from this project will therefore be an evaluation tool and a guide for documenting activities that can contribute to evaluation. The evaluation tool will include modules for qualitative assessment based on semi-structured interviews of CoP participants; a survey tool of key indicators of CoP performance within several domains and that can be scored quantitatively; and a more formal quantitative social network analysis. To develop these tools, the project is prospectively observing and documenting two newly formed entities that we believe will evolve into more sustainable communities of practice: one of these is engaged in practice guideline development activities from a national perspective using an adaptation model[44], and the other is a newly formed national community of academically-based and community-based surgeons engaged in developing synoptic reporting templates for cancer surgery[45]. In addition, the project is collaborating with the Partnership's knowledge management team to design a tool for monitoring virtual communities of practice created through access to a common web-based collaborative site. The online collaborative site may be a potential vehicle for facilitating collaborative efforts among different types of communities of practice. Finally, the project is working with an already established community of practice of oncology nurses for developing guidelines in supportive cancer care.

The CoP Project should help test in the Canadian health care context Wenger concept and the claim that communities of practice provide a concrete organizational infrastructure for realizing the dream of a learning organization [1]. However, in the course of generating and scaling our list of indicators in preparation for field testing the project toolkit, it became apparent that adhering to the integrity of Wenger's concept did not go very far in diagnosing what makes collaborative arrangements work, or not work. Missing from the concept are statements about *how *group activities are negotiated. The decision was thus made to collect a wider range of data on matters relating to group conflict and how differences of opinion are resolved. In so doing, we hope to generate a more robust account of what constitutes successful collaboration, particularly in the context of groups of health professionals who must find adept and credible means to interpret the wealth of research evidence in their respective fields and generate consensus on recommendations for practice. Results from the CoP Project will be published in 2010.

Notwithstanding some limitations to realizing Wenger's concept, communities of practice models have been recently and successfully adopted in Canadian health care [6,13,26,29]. One instance is in the province of Alberta, where a communities of practice model was used to optimize inter-professional communication and patient safety across three large health care regions in the province [6]. Each community of practice was allowed to choose a topic on which to focus, and to determine what was important for that topic. This autonomy created a sense of ownership and buy-in among participants, thus leading White *et al*. to conjecture that the changes implemented by the groups--such as streamlined admission and discharge processes for patients, and improved inter-professional communication--are likely to persist. A second example involves the current Surgical Oncology Program at Cancer Care Ontario, which adapted Wenger's concept to facilitate quality improvements in cancer surgery in Ontario. Adaptation included integrating the concept with other theories, such as health care improvement methodology and Nonaka's knowledge creation theory, as well as providing considerable management support to the groups and tying professional accreditation incentives to practitioners' involvement in improvement initiatives [13]. Preliminary results are encouraging, and show high levels of satisfaction with clinical leadership and knowledge translation among participating physicians. Results from this latter study suggest that Wenger's concept may be a supple starting point to effective collaboration in Canadian health care.

## Summary

Wenger's concept has been very successful in highlighting the importance of learning from peers and through practice, and in generating high levels of job satisfaction among participants. It is important to continue to evaluate Wenger's concept rigorously in order to open its possibilities and understand its limitations within an accountability framework. To do this, we have started through this discussion and in the Partnership's CoP Project initiative to put pressure on various aspects of Wenger's concept, such as the terms *organic *and *community*, to determine the sort of work they must perform in his model of collaboration. In doing so, we hope to establish an informed nomenclature around communities of practice, to advance the field of knowledge translation by bringing research and theory together, and to sharpen the conceptual tools decision makers and health care professionals need to foster professional learning and improvements across their organizations and regions.

## Competing interests

The authors declare that they have no competing interests.

## Authors' contributions

CB, GB, and BP developed the conceptual framework. CB and BP participated in the literature review. CB drafted the manuscript. All authors provided comments and approved the final manuscript.

## Pre-publication history

The pre-publication history for this paper can be accessed here:

http://www.biomedcentral.com/1472-6963/10/3/prepub

## References

[B1] Wenger E McDermott R Snyder WM Cultivating communities of practice 2002 Boston, Harvard Business School Press

[B2] Wenger E Communities of practice: the organizational frontier Harvard Business School Press 2000 January-February 139 145

[B3] Lave J Wenger E Situated learning: legitimate peripheral participation 1991 New York, Cambridge UP

[B4] Pereles L Lockyer J Fidler H Permanent small groups: group dynamics, learning, and change J Contin Educ Health Prof 2002 22 4 205 213 10.1002/chp.1340220404 12613055

[B5] Parboosingh JT Physician communities of practice: where learning and practice are inseparable J Contin Educ Health Prof 2002 22 4 230 236 10.1002/chp.1340220407 12613058

[B6] White D Suter E Parboosingh IJ Taylor E Communities of practice: creating opportunities to enhance quality of care and safe practices Healthc Q 2008 11 3 Spec 80 84 18382166 10.12927/hcq.2008.19654

[B7] Brown JS Duguid P Organizational learning and communities-of-practice: toward a unified view of working, learning, and innovation Organization Science 1991 1 1 40 57 10.1287/orsc.2.1.40

[B8] Nonaka I Takeuchi H The knowledge creation company: how Japanese companies create the dynamics of innovation 1995 New York, Oxford UP

[B9] Senge PM The fifth discipline: the art and practice of the learning organization 1990 New York, Doubleday

[B10] Newell S Edelman L Scarbrough H Swan J Bresnen M 'Best practice' development and transfer in the NHS: the importance of process as well as product knowledge Health Serv Manage Res 2003 16 1 1 12 10.1258/095148403762539095 12626022

[B11] Iedema R Meyerkort S White L Emergent modes of work and communities of practice Health Serv Manage Res 2005 18 1 13 24 10.1258/0951484053051906 15807977

[B12] Andrew N Tolson D Ferguson D Building on Wenger: communities of practice in nursing Nurse Educ Today 2008 28 2 246 252 10.1016/j.nedt.2007.05.002 17599697

[B13] Fung-Kee-Fung M Goubanova E Sequeira K Abdulla A Cook R Crossley C Langer B Smith AJ Stern H Development of communities of practice to facilitate quality improvement initiatives in surgical oncology Qual Manag Health Care 2008 17 2 174 185 18425031 10.1097/01.QMH.0000316995.79167.be

[B14] Gabbay J Le May A Jefferson H Webb D Lovelock R Powell J Lathlean J A case study of knowledge management in multiagency consumer-informed 'communities of practice': implications for evidence-based policy development in health and social services Health(London) 2003 7 3 283 310 10.1177/1363459303007003003

[B15] Carlile PR Transferring, translating, and transforming: an integrative framework for managing knowledge across boundaries Organization Science 2004 15 5 555 568 10.1287/orsc.1040.0094

[B16] Black PE Plowright D Exploring pharmacists' views about the contribution that reflective learning can make to the development of professional practice International Journal of Pharmacy Practice 2007 15 June 149 155 10.1211/ijpp.15.2.0011

[B17] Bartunek J Trullen J Bonet E Sauquet A Sharing and expanding academic and practitioner knowledge in health care J Health Serv Res Policy 2003 8 Suppl 2 62 68 10.1258/135581903322405199 14596750

[B18] Kerfoot K The leader as chief knowledge officer Nurs Econ 2002 20 1 40 41 11892548

[B19] Lathlean J Le May A Communities of practice: an opportunity for interagency working J Clin Nurs 2002 11 3 394 398 10.1046/j.1365-2702.2002.00630.x 12010537

[B20] Fish SE Interpreting the *Variorum *Critical Inquiry 1976 2 3 465 405 10.1086/447852

[B21] Barwick M Developing a community of practice model for cancer and chronic disease prevention 2008 Toronto, Ontario, Canada

[B22] Bate SP Robert G Knowledge management and communities of practice in the private sector: lessons for modernizing the national health service in England and Wales Public Administration 2002 80 4 643 663 10.1111/1467-9299.00322

[B23] Wenger E Knowledge management as a doughnut: shaping your knowledge strategy through communities of practice Ivey Business Journal Online 2004

[B24] Chua AYK The rise and fall of a community of practice: a descriptive case study Knowledge and Process Management 2006 13 2 120 128 10.1002/kpm.239

[B25] Fung-Kee-Fung M Watters J Crossley C Goubanova E Abdulla A Stern H Oliver TK Regional collaborations as a tool for quality improvements in surgery: a systematic review of the literature Annals of Surgery 2009 249 4 565 572 10.1097/SLA.0b013e31819ec608 19300234

[B26] Fung-Kee-Fung M Morash R Goubanova E Champlain regional cancer surgery program annual CoP survey report 2007 Ottawa, Ontario, Canada

[B27] Bassi S Polifroni EC Learning communities: the link to recruitment and retention J Nurses Staff Dev 2005 21 3 103 109 10.1097/00124645-200505000-00005 15940026

[B28] Russell J Greenhalgh T Boynton P Rigby M Soft networks for bridging the gap between research and practice: illuminative evaluation of CHAIN BMJ 2004 328 7449 1174 10.1136/bmj.328.7449.1174 15142924 PMC411097

[B29] Norman CD Heurta T Knowledge transfer and exchange through social networks: building foundations for a community of practice within cancer control Implementation Science 2006 1 20 16999871 10.1186/1748-5908-1-20 PMC1599751

[B30] Tetroe JM Graham ID Foy R Robinson N Eccles MP Wensing M Durieux P Legare F Nielson CP Adily A Ward JE Porter C Shea B Grimshaw JM Health research funding agencies' support and promotion of knowledge translation: an international study Milbank Q 2008 86 1 125 155 10.1111/j.1468-0009.2007.00515.x 18307479 PMC2690338

[B31] Gagliardi A Ashbury FD George R Irish J Stern HS Improving cancer surgery in Ontario: recommendations from a strategic planning retreat Canadian Journal of Surgery 2004 47 4 270 276 PMC3211788 15362329

[B32] Mittman BS Creating the evidence base for quality improvement collaboratives Ann Intern Med 2004 140 11 897 901 15172904 10.7326/0003-4819-140-11-200406010-00011

[B33] Fox S Communities of practice, Foucault and actor-network theory Journal of Management Studies 2000 37 6 853 867 10.1111/1467-6486.00207

[B34] Marshall N Rollinson J Maybe Bacon had a point: the politics of interpretation in collective sensemaking British Journal of Management 2004 15 S71 S86 10.1111/j.1467-8551.2004.00407.x

[B35] Swan J Scarbrough H The politics of networked innovation Human Relations 2005 58 7 913 943 10.1177/0018726705057811

[B36] Roberts J Limits to Communities of Practice Journal of Management Studies 2006 43 3 623 639 10.1111/j.1467-6486.2006.00618.x

[B37] Granovetter M The strength of weak ties American Journal of Sociology 1973 78 1360 1380 10.1086/225469

[B38] Coopey J Burgoyne J Politics and organizational learning Journal of Management Studies 2000 37 6 869 885 10.1111/1467-6486.00208

[B39] Li L Grimshaw JM Nielsen C Judd M Coyte PC Graham ID Evolution of Wenger's concept of community of practice Implementation Science 2009 4 11 10.1186/1748-5908-4-11 PMC2654669 19250556

[B40] Knowledge sharing communities and networks http://www2.warwick.ac.uk/fac/soc/wbs/projects/kcn/ph1/

[B41] Anderson B Imagined communities: reflections on the origins and spread of nationalism 1983 London UK, Verso

[B42] Wenger E Communities of practice: learning, meaning, and identity 1998 Cambridge, Cambridge UP

[B43] D'Amour D Goulet L Labadie JF San Martin-Rodriguez Pineault R A model and typology of collaboration among professionals in healthcare organizations BMC Health Services Research 2008 8 188 18803881 10.1186/1472-6963-8-188 PMC2563002

[B44] Fervers B Burgers JS Haugh MC Latreille J Mlika-Cabanne N Paquet L Coulombe M Pooirier M Burnand B Adaptation of clinical guidelines: literature review and proposition for a framework and procedure Int J Qual Health Care 2006 18 3 167 176 10.1093/intqhc/mzi108 16766601

[B45] Edhemovic I Temple WJ de Gara CJ Stuart GCE The computer synoptic operative report--A leap forward in the science of surgery Ann Surg Oncol 2004 11 10 941 947 10.1245/ASO.2004.12.045 15466354

